# Intra-orbital Marble Injury in a Child After a Fall: A Case Report

**DOI:** 10.7759/cureus.82749

**Published:** 2025-04-21

**Authors:** Nur Fatihin Samiyah Mohamad Hisham, Othmaliza Othman, Akmal Haliza Zamli

**Affiliations:** 1 Ophthalmology, Universiti Kebangsaan Malaysia Medical Center, Kuala Lumpur, MYS; 2 Ophthalmology, Hospital Tengku Ampuan Afzan, Kuantan, MYS

**Keywords:** intra-orbital foreign body, laceration, marble, pediatric, polyvinyl chloride

## Abstract

An intra-orbital foreign body is a devastating ocular condition that might end up as blindness if prompt treatment is not carried out. This is a case of a 2-year-old girl who presented with a right lower eyelid laceration. She was playing with a marble and a polyvinyl chloride (PVC) pipe. Accidentally, she fell with the PVC pipe that pierced her right lower eyelid region. CT scan of the brain, orbit, and paranasal sinus revealed a spherical-shaped high-attenuation focus within the right maxillary sinus extending to the inferior orbital cavity. She underwent surgical exploration and removal of the foreign body and recovered well after surgery.

Pediatric periocular injury demands a high index of suspicion for a more serious injury, such as an intra-orbital foreign body. A thorough history, examination, and appropriate imaging are crucial in such cases. A meticulous removal of the foreign body is important to avoid complications and injury to surrounding structures.

## Introduction

An intra-orbital foreign body is defined as any foreign object or particle that is embedded within the eyeball or orbital cavity. The injury could be a high-impact or even a trivial injury that could steal the vision [1]. Intra-orbital foreign bodies are able to gain access to the orbit by an open injury between the eyeball and the orbital walls, and even from the paranasal sinus [1]. A foreign body that lodges inside the orbital cavity could impinge on the nearby structures, causing injury to the globe, the extraocular muscles, and the cranial nerves.

When a patient exhibits ocular trauma, conducting a comprehensive history and physical examination, which includes inspecting for entry wounds, is crucial. Physicians must promptly assess to exclude more serious injury, such as penetrating injury, globe rupture, and the possibility of optic nerve trauma. A high index of suspicion is important to exclude a retained intra-orbital foreign body, especially in trivial injury, particularly in cases that occurred in children who are at their pre-verbal age, who are unable to give a clear history, and the incident is unwitnessed. The outcome of eye trauma in childhood would be worse compared to adults due to immaturity of the visual function and increasing their potential to develop amblyopia [2].

## Case presentation

**Figure 1:**
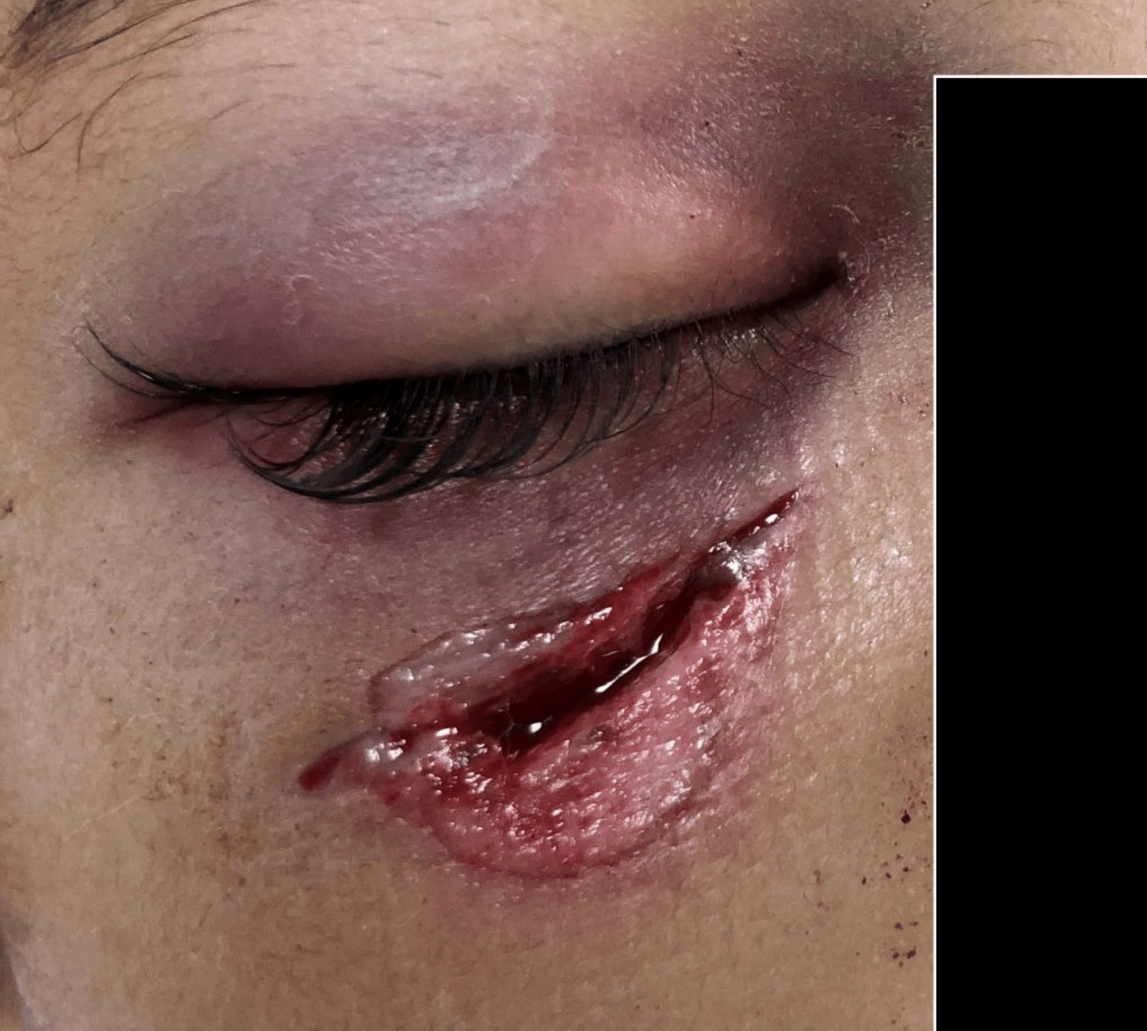
Clinical appearance of the right lower lid laceration wound, post-trauma

Our case is of a 2-year-old child who was fortunate that her parents immediately brought her to an emergency department at one of the primary centers near their area. Imaging was not performed, and she was referred to the emergency department of our tertiary center after she was found to have a lower lid laceration wound after playing with a polyvinyl chloride (PVC) pipe alone in her house. According to her mother, the incident was unwitnessed, but beforehand, the child was seen playing with a single-ended PVC pipe containing a small marble within it. She accidentally fell on her face in which there was a direct hit involving one end of the PVC pipe. Following the injury, the child was crying in pain, and her mother applied a pressure bandage over the laceration wound, and subsequently, the bleeding stopped. Otherwise, the child was alert and conscious with no history suggestive of head injury such as headache, nausea, or vomiting. However, a small but deep wound was found 2 cm below the right lower eyelid (Figure 1), and thus she was referred to the Plastic Surgery Department for toilet suturing of the right lower eyelid laceration.

**Figure 2:**
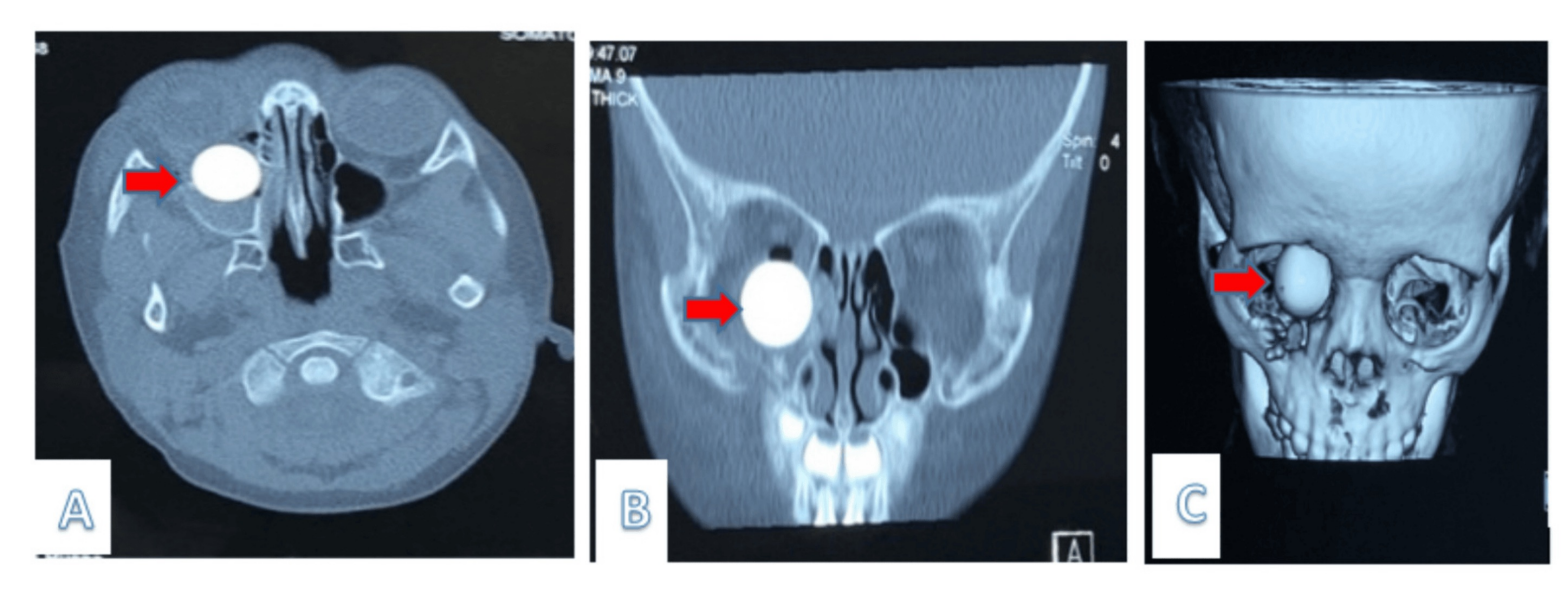
(A) Axial and (B) coronal (C) reconstruction view of a CT scan showing a foreign object spherical in shape, measuring 1.6 (AP) x 1.6 (W) cm, and situated in the right maxillary sinus AP: anteroposterior, W: width

Upon arrival to the emergency department, she was urgently posted for a CT of the orbits and paranasal sinuses. The imaging clearly exposed a high-density spherical-shaped foreign object measuring 1.6 cm in height, anteroposterior, and width within the right side of the maxillary sinus, and it extended towards the inferior orbital cavity. Assuming that it was a high-impact injury, the right maxillary sinus was also found to have a comminuted fracture as well as the medial and inferior orbital walls. Both globes are seen intact. However, there is a right exophthalmos with right pneumo-orbital. The optic nerve is preserved. The right inferior rectus muscle is seen displaced superiorly and laterally by the focus (Figure 2).

Thus, the patient was referred to the ophthalmology team to collaborate with the otorhinolaryngology (ORL) team for surgical exploration and removal of the foreign body.

Performing the ocular examination proved challenging due to the patient's lack of cooperation. Initially, we were unable to determine visual acuity, although the patient's right eye appeared unresponsive to bright light. Fortunately, the relative afferent pupillary defect was negative. Additionally, there was a restriction in the elevation of the right eye and also proptosis.

**Figure 3:**
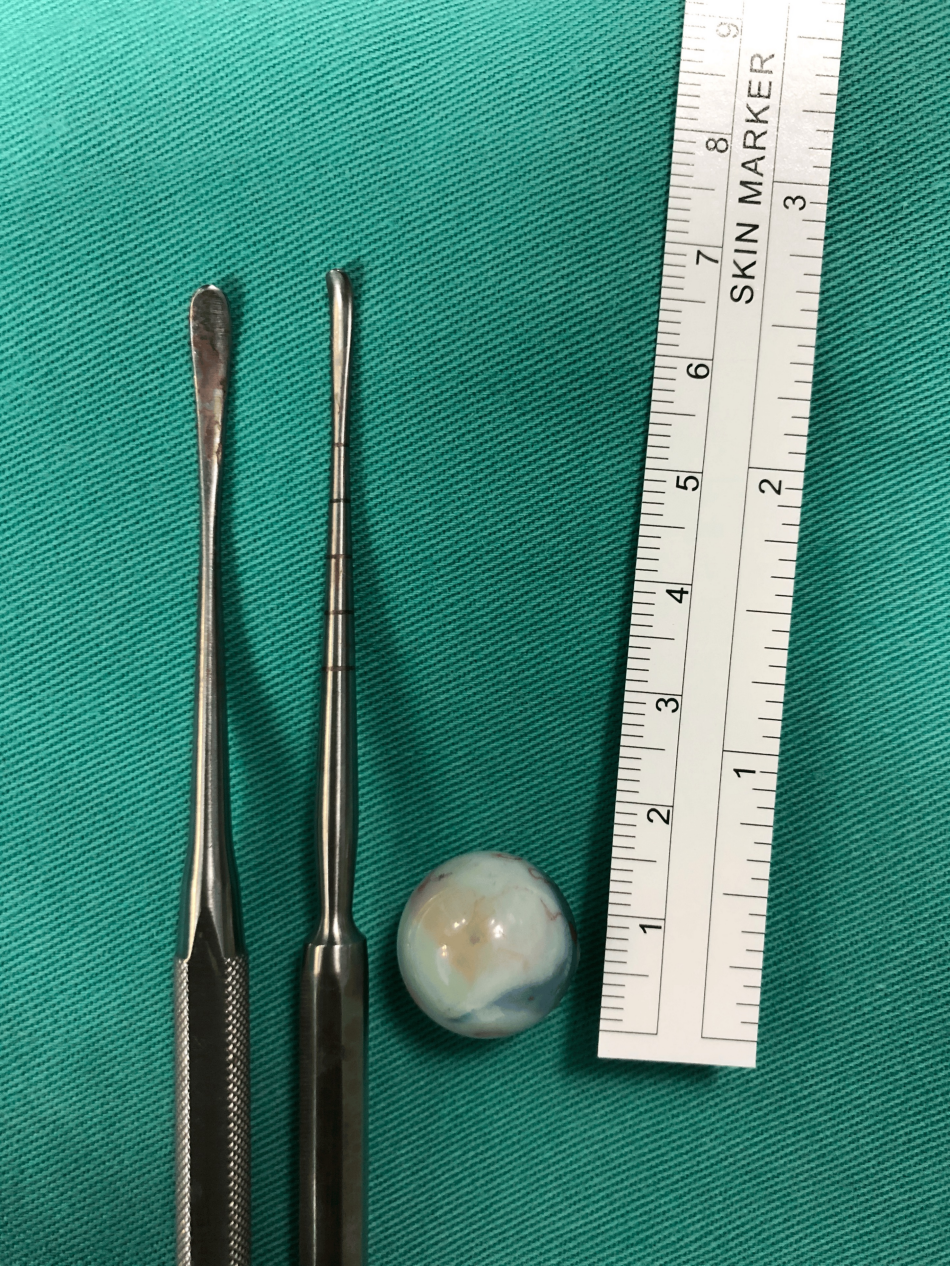
Freer periosteal elevator used intraoperatively to retrieve the foreign body, which measured 1.5 cm in diameter

Wound exploration revealed a long wound extending towards the inferomedial floor of the orbit with an embedded round white foreign body. Endoscopically, the tip of the foreign body was visible at the superomedial roof of the maxillary sinus. The foreign body was successfully removed through the entry wound using two Freer periosteal elevators with the aid of endoscopic view performed by the ORL team (Figure 3).

The entry wound measured 2 cm horizontally, the width of 0.5 cm situated 1 cm below the right inferior lid margin, and was closed in layers. The relative afferent pupillary defect was negative throughout surgery. The patient was given a course of systemic antibiotics: amoxicillin, clavulanic acid, and metronidazole.

On day 1 post-operation, right eye vision was 6/12 and left eye was 6/9. There was a right mechanical ptosis; the anterior segment was unremarkable. The patient was noted to have a slight limitation upon right eye elevation. There was Berlin’s edema upon fundus examination of the right eye. The patient was discharged on day 3 post-operation. Upon review on day 10 post-operation, vision over the right eye improved to 6/9, and extraocular muscle movement over the right eye was full. The entry wound was well healed.

## Discussion

Trauma to the eyeball or orbit during childhood is usually caused by penetrating and blunt injury that results from falls and bumps that occur during games or sports. A trauma may result in varying degrees of injury to the eye, despite a trivial one, and may cause irreversible blindness in pediatrics, which requires immediate attention [2,3].

The severity of injury in penetrating trauma of the orbit is often underestimated in a physical examination and could be missed [1], especially when it involves the pediatric age group.

Upon attending cases that come due to ocular trauma, a diligent physician should be able to elicit a more serious complication from the injury, such as retained foreign material, especially in cases where the injury is trivial and the presentation only reveals the exposed injury, where imaging is very useful for assessment of the nearby structures that cannot be assessed during clinical examination. Herein, proper history taking regarding the mechanism of injury and a high index of suspicion would be a clue, especially in a pre-verbal child where the injury is unwitnessed [3,4].

Radiological imaging, especially CT scan, is extremely useful in the detection of foreign material or objects, and a very helpful tool in localizing the object, evaluating the surrounding tissue, and assisting in a preoperative plan to ensure successful surgery and minimize complications [5]. This imaging helped identify associated fractures and displaced orbital structures, which were critical to minimizing intraoperative risks.

The successful management of this case underscores the importance of multidisciplinary collaboration. The combined use of endoscopic visualization and direct surgical removal through the entry wound represents an effective and minimally invasive approach. This technique reduced the need for more extensive surgical interventions, thus minimizing potential complications, recovery time, and cosmetic impacts. The use of a Freer periosteal elevator for the retrieval of the marble highlights the value of employing versatile surgical tools in narrow anatomical spaces. This approach allowed for controlled manipulation and extraction without causing additional trauma to surrounding structures. The approach in this case was tailored to the child’s anatomical and cooperative limitations, avoiding prolonged general anesthesia or unnecessary incisions. This highlights the importance of adapting surgical strategies based on patient-specific factors.

Parent and caretaker supervision to ensure no hazardous tools are in the house is extremely important to prevent trauma from occurring because this is the major contribution to ocular trauma cases in the pediatric group [6].

## Conclusions

Pediatric periocular injury demands a high index of suspicion for a more serious injury, such as an intra-orbital foreign body. A thorough history, examination, and appropriate imaging are crucial in such cases. The surgical technique employed, involving minimally invasive intervention through the entry wound with endoscopic guidance, ensured precise removal of the foreign body while minimizing complications and preserving function. This case underscores the need for individualized surgical planning tailored to patient-specific anatomical and clinical factors, ensuring optimal recovery with minimal cosmetic and functional sequelae. Nonetheless, awareness regarding eye injuries among the pediatric age group has to be emphasized, especially among parents and caregivers, because prevention is better than cure.
